# Nutraceuticals and Hypertensive Disorders in Pregnancy: The Available Clinical Evidence

**DOI:** 10.3390/nu12020378

**Published:** 2020-01-31

**Authors:** Silvia Fogacci, Federica Fogacci, Arrigo F.G. Cicero

**Affiliations:** 1 Medical and Surgical Sciences Department, Sant’Orsola-Malpighi University Hospital, Building 2-IV Floor, Via Albertoni 15, 40138 Bologna, Italy; silviafogacci@alice.it (S.F.); federicafogacci@gmail.com (F.F.); 2 Italian Nutraceutical Society (SINut), Via Guelfa 9, 40138 Bologna, Italy

**Keywords:** pregnancy, hypertension, hypertensive disorders, pre-eclampsia

## Abstract

The aim of the present critical review is to summarize the available clinical evidence supporting the use of some dietary supplements that have been shown to lower blood pressure in hypertensive pregnant women. A systematic search strategy was carried out to identify trials in MEDLINE (National Library of Medicine, Bethesda, Maryland, MD, USA; January 1980 to September 2019) and the Cochrane Register of Controlled Trials (The Cochrane Collaboration, Oxford, UK). The terms ‘nutraceuticals’, ‘dietary supplements’, ‘pregnancy’, ‘pre-eclampsia’, ‘clinical trial’, and ‘human’ were incorporated into an electronic search strategy. The references of the identified studies and review articles were reviewed to look for additional studies of interest. We preferably selected papers that reported recent comprehensive reviews or meta-analysis, or original clinical trials of substances with blood pressure-lowering or vascular protective effect in pregnancy. There is a relative body of evidence that supports the use of calcium, vitamin D, folic acid, and resveratrol in preventing the development of hypertensive disorders in pregnancy, and evidence supporting drug treatment too. Further clinical research is advisable to identify the dosage and timing of the supplementation, the group of women that might benefit the most from this approach, and the nutraceuticals with the best cost-effectiveness and risk-benefit ratio for widespread use in clinical practice.

## 1. Background

Hypertensive disorders in pregnancy are among the most common medical complications, affecting 5% to 10% of pregnancies worldwide [1]. These disorders include chronic hypertension, gestational hypertension, pre-eclampsia, and chronic hypertension with superimposed pre-eclampsia, being a major cause of maternal, fetal, and neonatal morbidity and mortality [2].

Evidence-based data regarding treatment of hypertension during pregnancy are lacking, and there is no evidence supporting what target blood pressure values should be reached [3]. The management of hypertension in pregnancy depends on blood pressure levels, gestational age, and presence of associated maternal and fetal risk factors. However, almost all drugs recommended by the latest international guidelines have a questionable safety profile for the fetus [4,5], so that the risk–benefit ratio of this treatment should always be carefully considered in clinical practice for each individual.

Although non-pharmacological treatments have always been considered marginal in the management of hypertension in pregnancy, their role should be taken into account in light of the most recent evidence. As a matter of fact, randomized controlled clinical trials testing the efficacy and safety of dietary supplements in pregnancy are numerous compared to conventional drugs, and their results seem to be promising [6]. For this reason, their use in clinical practice should be encouraged [7].

**Figure 1:**
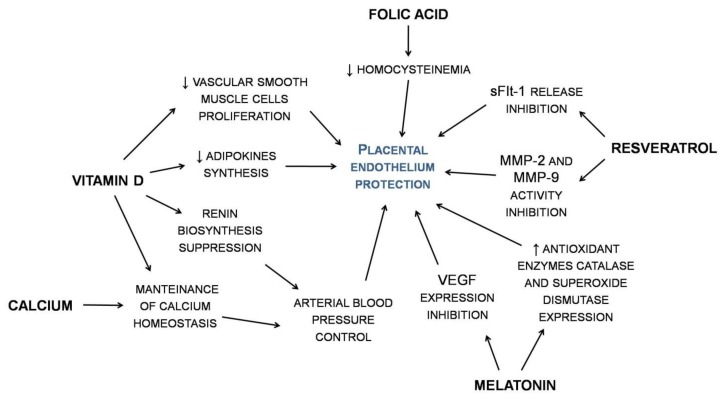
Selected bioactive compounds with demonstrated blood pressure lowering effects in hypertensive disorders in pregnancy. MMP—matrix metalloproteinase; sFIt1—soluble fms-like tyrosine kinase-1; VEGF—vascular endothelial growth factor.

With these premises, the aim of the present critical review was to summarize the available clinical evidence supporting the use of some bioactive compounds with demonstrated blood pressure lowering effects in pregnancy (Figure 1).

## 2. Methods

For the purpose of this review, a systematic search strategy was carried out to identify trials in both MEDLINE (National Library of Medicine, Bethesda, Maryland, MD, USA; January 1980 to September 2019) and the Cochrane Register of Controlled Trials (The Cochrane Collaboration, Oxford, UK). The terms ‘nutraceuticals’, ‘dietary supplements’, ‘pregnancy’, ‘high blood pressure’, ‘hypertension’, ‘pre-eclampsia’, ‘preeclampsia’, ‘clinical trial’, and ‘human’ were incorporated into an electronic search strategy. The references of the identified studies and review articles were reviewed to look for additional studies of interest. The authors reviewed all of the citations retrieved from the electronic search to identify potentially relevant articles for this review. We excluded in vitro data, animal studies and studies focused on human data, in order to limit our report to food components and nutraceuticals for which safety and tolerability in humans are already known. Therefore, we preferably selected papers reporting recent comprehensive reviews or meta-analyses, or original clinical trials on substances with a blood pressure-lowering or vascular protective effect in pregnancy.

## 3. Results

A relative number of nutraceutical compounds are known for having a beneficial effect in the management of hypertension and hypertensive disorders in pregnancy. In this review, we will focus on calcium, vitamin D, folic acid, resveratrol, sodium and potassium, zinc, and melatonin, which encounter greater evidence specific to pregnancy.

### 3.1. Calcium

An adequate calcium intake during pregnancy reduces the risk of hypertensive disorders while pregnant [8]. Furthermore, calcium supplementation is attractive as a potential intervention to reduce the risk of developing pre-eclampsia, as it has good bioavailability when administered in the form of carbonate, citrate, lactate, or gluconate [8,9].

A retrospective study involving 11,387 pregnant women showed that daily supplementation of 500 mg of oral calcium for more than 6 months during pregnancy is associated with a 45% reduction in the risk of developing hypertension [Relative Risk (RR) = 0.55, 95%CI: 0.33; 0.93] [10]. A meta-analysis by the Cochrane Collaboration showed that calcium supplementation compared to placebo decreased the overall risk of pre-eclampsia in pregnancy (*n* = 15,730 women; RR = 0.45, 95%CI: 0.31; 0.65), with an even greater reduction in women clinically diagnosed at high risk (*n* = 587 women; RR = 0.22, 95%CI: 0.12; 0.42) [11]. Similar results were confirmed in a more recent meta-analysis of 27 clinical studies, involving a total of 28492 pregnant women (RR = 0.51, 95%CI: 0.40; 0.64) [12].

An adequate calcium intake might also help to avoid superimposed pre-eclampsia in patients with resistant hypertension [13]. The mechanism by which calcium may have an effect on blood pressure is still unclear; one hypothesis is that low calcium intakes increase the levels of parathyroid hormone and 1,25-dihydroxy vitamin D, which are required to maintain specific calcium concentrations in extracellular fluids. Higher levels of parathyroid hormone and 1,25-dihydroxy vitamin D stimulate calcium influx into different cell types and increase intracellular calcium influx into the vascular smooth muscle cell, and consequently increase muscle reactivity, peripheral vascular resistance, and thus, raise blood pressure [14].

However, some concerns have been raised regarding the safety profile of calcium supplementation during gestation, as it may cause rebound postnatal bone demineralization and it is thought to increase the occurrence of HELLP syndrome, which involves HEmolysis, elevated Liver enzymes, and a Low Platelet count [15].

The most recent European Society of Cardiology (ESC), American College of Obstetricians and Gynaecologists (ACOG), and World Health Organization (WHO) guidelines recommend calcium supplementation to be prescribed in deficiency (< 600 mg/day) during pregnancy to reduce the risk of pre-eclampsia [4,5,8]. In this case, the suggested scheme for calcium supplementation is 1.5–2.0 g daily, with the total dosage divided into three dosages, preferably taken at mealtimes. Negative interactions may occur with the simultaneous supplementation of iron and calcium, which is why the two micronutrients should preferably be administrated several hours apart [8].

### 3.2. Vitamin D

Vitamin D deficiency, as measured by circulating 25(OH)-vitamin D concentrations, is reported to be as high as 40% among pregnant women and is also very common during lactation [16]. In Mediterranean countries, where vitamin D deficiency is even more prevalent (up to 60% to 80%), vitamin D supplementation and policies of food fortification are currently not recommended during pregnancy, and they are missing from clinical practice [17]. As pregnancy progresses, the requirement for vitamin D increases and as a consequence, any pre-existing vitamin D deficiency can worsen. Vitamin D supplementation was demonstrated to potentiate nifedipine treatment for pre-eclampsia, shortening the time to control blood pressure and prolonging the time before subsequent hypertensive crisis, probably via an immunomodulatory mechanism [18].

A recent meta-analysis carried out on 4777 women suggested that treatment with vitamin D reduced the risk of pre-eclampsia compared to no intervention or placebo [Odd Ratio (OR) = 0.37, 95%CI: 0.26; 0.52], the effect being largely independent of the supplementation duration and being enhanced according to increasing vitamin D doses. Based on these data, the supplementation of around 25000 UI/week of vitamin D is recommended from the first trimester of gestation, along with monitoring for calcemia and calciuria as markers of potential vitamin D overdose [19].

Adequate vitamin D intake might help with the maintenance of calcium homeostasis, which is inversely related to blood pressure levels, or may directly suppress the proliferation of vascular smooth muscle cells [20]. Furthermore, vitamin D might be a powerful endocrine suppressor of renin biosynthesis and could impact on the regulation of the renin-angiotensin system, which plays a critical role in blood pressure control [20]. Finally, vitamin D may impact on the synthesis of adipokines related to endothelial and vascular health [21].

### 3.3. Folic Acid

Epidemiological studies of the association between folic acid supplementation and the incidence of pre-eclampsia showed a potential protective effect. Findings from the Ottawa and Kingston (OaK) Birth Cohort suggested a 60% reduction in the risk of pre-eclampsia (*n* = 8085; OR = 0.37, 95%CI: 0.18; 0.75) and a dose-response association between folic acid and pre-eclampsia events in women with identified risk factors [22]. A potential reason can be found in folic acid action affecting levels of hyperhomocysteinemia, which is known to damage the vascular endothelium of the developing placenta [22]. Moreover, a folate deficiency may induce the apoptosis of human cytotrophoblast cells, possibly affecting trophoblast invasion and placental development [23].

In a randomized clinical trial of supplementation with a multivitamin containing 0.8 mg of folic acid in relation to hypertension in pregnancy in a high-risk population of women positive for antibodies to HIV, a 38% reduction was observed in the primary composite outcome of gestational hypertension (including pre-eclampsia or eclampsia) in the intervention group compared to the placebo group [24]. Other forms of folate, including 5-methyltetrahydrofolate, were investigated and had similar results, whereas folic acid antagonists showed the opposite effect, increasing the risk of pre-eclampsia [25].

A meta-analysis of 13 cohort studies and one randomized clinical trial, overall involving 309882 pregnant women, showed that supplementation with multivitamins containing folic acid during pregnancy significantly lowers pre-eclampsia risk (RR = 0.69, 95%CI: 0.58; 0.83) but not the risk of gestational hypertension (RR = 1.19, 95%CI: 0.92; 1.54) [26]. However, the Folic Acid Clinical Trial (FACT) recently failed to demonstrate that supplementation with 4 mg/day folic acid beyond the first trimester of gestation prevents pre-eclampsia in women at high risk for this condition (*n* = 2464; RR = 1.10, 95%CI: 0.90; 1.34) [27].

Further studies are needed to clarify in which group of women (in the exposed group, in the non-exposed one, or in both of them) a folic acid supplementation is necessary in order to reduce the risk of pre-eclampsia. Moreover, the dosage and timing of this supplementation are still unclear.

### 3.4. Resveratrol

Resveratrol (3,5,40-trihydroxystilbene) belongs to a family of polyphenolic compounds known as stilbenes, which are particularly concentrated in grapes and red wine [28]. Despite the amount of resveratrol in foods, its bioavailability after oral administration is usually poor in humans (<1%) in consideration of biotransformation phenomena that happen in the liver microsomes and intestine and lead to less active or completely inactive metabolites from intestinal bacterial metabolism [29].

The anti-hypertensive effects of resveratrol were shown in several pre-clinical models of hypertension, through many mechanisms involving its anti-oxidant properties, the stimulation of nitric oxide endothelial production, the inhibition of vascular inflammation, and the prevention of platelet aggregation [29]. However, the short half-life of this molecule and its labile properties, rapid metabolism and elimination restrict the potential therapeutic application of resveratrol [30].

Findings from a recent meta-analysis show that the combination treatment of nifedipine and resveratrol is able to shorten, in pregnancy, the time for achieving target blood pressure (BP) with a relative risk (RR) reduction of −13.9 (95%CI: −22.6; −5.2), compared to nifedipine alone (RR = −3.5, 95%CI: −26.5; 19.7) and labetalol (RR = −1, 95%CI: −22.2; 23) [31], which are the recommended treatments from the International Guidelines for the management of pre-eclampsia [4,5].

As a matter of fact, it is reported that resveratrol could inhibit the release of soluble fms-like tyrosine kinase (sFlt-1) from the human placenta, which is known as a reliable biomarker for clinical prediction of pre-eclampsia [32]. Furthermore, a large body of evidence supports resveratrol’s action in regulating plasma levels and activities of matrix metalloproteinase (MMP)-2 and MMP-9 [33], which are also reported to be involved in pre-eclampsia, both as predictive biomarkers and drug targets [34]. Finally, resveratrol may have a very positive impact on blood pressure in pregnancy.

### 3.5. Sodium and Potassium

Recent findings suggested that pregnant women with pre-eclampsia with high dietary salt and low potassium intake had a greater maternal morbidity risk compared to those with pre-eclampsia under low dietary salt and high potassium intake [35]. An analysis from the Odense Child Cohort showed that salt intake >6 g/d in pregnancy is associated with a greater risk of developing pre-eclampsia (hazard ratio: 5.68, 95%CI: 1.51; 21.36) [36]. A further analysis from the Danish National Birth Cohort (DNBC) carried out on 66,651 singleton pregnancies from 62,774 women concluded that lower sodium intake in the second trimester of gestation is related to a reduced risk of hypertensive disorders in pregnancy. In particular, in the DNBC cohort, women with the highest sodium intake (median 3.70 g/day (range: 3.52–7.52 g/day)) had a 54% (95%CI: 16%; 104%) higher risk of gestational hypertension and a 20% (95%CI: 1%; 42%) higher risk of pre-eclampsia than women with the lowest intake of sodium (median 2.60 g/day (range: 0.83–2.79 g/day)) [37].

Sodium intake in the early stages of pregnancy is crucial for physiologic extracellular volume expansion, which regulates maternal blood pressure and utero-placental circulation [38]. However, it is still unclear whether dietary salt has a causal association with the risk of hypertensive disorders in pregnancy. It is also unknown whether placental sodium metabolism is responsible for a lower volume expansion coupled with higher urinary sodium excretion observed in pre-eclampsia [39]. For this reason, further research that considers the role of sodium intake during pregnancy is needed.

### 3.6. Zinc

A low maternal circulating zinc concentration was associated with pregnancy complications, including pre-eclampsia [40]. The main factor that determines zinc status is diet. Grains and legumes contain a significant amount of phytic acid, and phytate binding of zinc limits its absorption in the small intestine, contributing to zinc deficiency [41]. Estimates based on the bioavailability of zinc, physiological requirements, and predicted zinc absorption suggest the prevalence of zinc deficiency to range from 4% in European countries, including the United Kingdom, Sweden, Germany, and France, to 73% in Bangladesh, India, and Nepal [42]. In the United States and Australia, an additional 2–4 mg of zinc per day is recommended to be supplemented in pregnant women compared to non-pregnant women [41,43]. It is widely acknowledged that many pregnant women do not meet this recommendation [44], particularly in developing countries where diets are often plant-based. A more recent evaluation predicts inadequate zinc intake in over 25% of populations in Southeast Asia and Africa [45].

However, attempts to modify the incidence of pre-eclampsia with zinc supplementation have not been successful. A meta-analysis from the Cochrane Collaboration Group involving seven randomized clinical trials with 2975 enrolled women failed to show that zinc supplementation might significantly decrease the risk of hypertension or pre-eclampsia (RR = 0.83, 95%CI: 0.64; 1.08) [46].

### 3.7. Melatonin

Physiologically, maternal melatonin levels gradually increase throughout gestation mainly due to placental production [47]. Melatonin easily crosses the placenta in order to enter the fetal circulation, and it is known to be important in promoting fetal growth and brain development while regulating placental homeostasis and hormone production [48]. Available information indicates that in the placenta melatonin induces the expression of the antioxidant enzymes catalase and superoxide dismutase, prevents injury caused by oxidative stress, and inhibits the expression of vascular endothelial growth factor (VEGF) [48].

In pregnancies complicated by pre-eclampsia, circulating maternal melatonin levels and placental melatonin receptor expression is significantly lower [49], probably due to a reduction in the activity of placental melatonin-synthesizing enzymes [50]. For this reason, women with pre-eclampsia are likely to benefit from melatonin supplementation even though evidence in this regard is still preliminary. In the PAMPR study, a sustained-release preparation of melatonin 10 mg given three times a day was shown to safely prolong pregnancy in women diagnosed with early-onset pre-eclampsia [51].

Further studies should be designed to evaluate the effect of melatonin on blood pressure levels and hypertensive disorders in pregnancy.

## 4. Discussion

During pregnancy, maternal and fetal outcomes are strongly influenced by the control of blood pressure [52,53]. While the maintenance of an adequate body weight is attainable through a balanced diet and physical activity, the anti-hypertensive treatment of hypertensive pregnant women is particularly complex, given the contraindication and the side effects of the most commonly used blood pressure-lowering drugs [4,5,6]. For these reasons, pharmacological treatment is only considered for the management of the most severe cases [4,5,6].

Based on the available evidence, the use of some nutraceuticals with safety profiles and a well-established effect in pregnancy might represent, alone or in combination with traditional drugs, a good therapeutic alternative to prevent and treat hypertensive disorders. Nevertheless, therapy needs to be carefully monitored and personalized, since dietary supplements consumed by the mother before or during fetal development seem to be able to affect the fetal epigenome [54].

Nutraceuticals currently encountering greater evidence in the treatment and prevention of hypertensive disorders during gestation are calcium, vitamin D, resveratrol and sodium/potassium, whereas treatment with folic acid, zinc and melatonin is only supported by preliminary data. In particular, several nutraceutical compounds with a clinically detectable effect in hypertensive disorders are also involved in glycemic control, providing a double metabolic advantage and making their use particularly attractive in pregnancy [55]. However, the poor bioavailability of some of these molecules in humans (e.g., resveratrol) limits their clinical application, and the development of new drug delivery systems intended to enhance their bioavailability might dramatically increase plasma levels and, presumably, their efficacy [28].

In general, sodium restriction and vitamin D supplementation should always be recommended in pregnancy for preventing hypertensive disorders and related problems. Otherwise, calcium is advised to be administrated only in the case of deficiency.

The use of nutraceuticals (e.g., vitamin D and resveratrol) with a good safety profile and well-established anti-hypertensive effect together with traditional drugs provides adequate blood pressure control and delays the time to relapse in pre-eclampsia, without exposing the mother and fetus to additional risks. Otherwise, the level of evidence indicating that folic acid, zinc and melatonin are effective in preventing hypertensive disorders and their complications is currently low.

The main difficulty in building recommendations that may implement the current international guidelines regarding the use of the above-cited dietary supplements in pregnancy is related to the limited number of available data, the lack of homogeneity among tested formulations and background diets of the enrolled patients, and gold-standard comparison problems. However, these conditions are very frequent when considering trials carried out on pregnant women and are mostly due to ethical concerns. On the other hand, these nutraceuticals have been largely tested in the general population, where the efficacy and safety profiles are clear. With regard to pregnant women, currently, we still need data on long-term safety regarding many of the above-discussed active compounds, particularly when they are supplemented at a high dosage or in combination with other nutraceuticals. Furthermore, the dosage and timing of the supplementation are often still unclear, as well as the specific molecular mechanisms underlying the observed effects.

Definitely, the available evidence suggests that some nutraceuticals are able to improve blood pressure control during pregnancy, also preventing some severe hypertension-related pregnancy complications. However, further clinical research is advisable to identify the nutraceuticals with the best cost-effectiveness and risk-benefit ratio for widespread use in clinical practice.
